# Utilization of post-fermentation sludge as a soil structure and strength conditioner

**DOI:** 10.1038/s41598-025-22871-w

**Published:** 2025-11-06

**Authors:** Angelika Gryta, Patrycja Boguta, Grzegorz Józefaciuk, Kamil Skic

**Affiliations:** Institute of Agrophysics, Polish Academy of Sciences, Doświadczalna 4 str, Lublin, 20-290 Poland

**Keywords:** Digestate, Post-fermentation sludge, Soil structure, Soil mechanical resistance, Model soil aggregates, Chemical physics, Environmental chemistry, Sustainability, Mechanical properties, Environmental impact

## Abstract

Available reports focus mostly on the effects of post-fermentation sludge (digestate) on soil organic carbon level, soil chemistry, and microbiology, and little is known about the impact on soil structural and mechanical properties. Therefore, the influence of different rates of digestate (1–15%) on the structure and strength of several soils, varying in grain size composition, pH, and organic matter content, was studied. The effects were analyzed by scanning electron microscopy, nitrogen adsorption, mercury porosimetry, bulk density, and mechanical stability tests. Organic sludge usually increased total porosity, average pore diameter, total pore volume, and diminished bulk density of all soil aggregates. Digestate addition significantly decreased the specific surface area of most clayed soils of the highest initial surface. The application of digestate increased the strength of initially most fragile sandy soil aggregates. The more intensive positive changes in the pore and surface characteristics and increase in mechanical strength of sandy soils highlighted the potential of the digestate application to enhance the stability and structure of less productive areas.

## Introduction

 The recent increase in human activity and related intensification of agriculture resulted in a significant reduction in soil organic matter (SOM) level on a large scale^1^ that accelerates soil susceptibility to erosion, compaction, and transport of pollutants into the aquatic environment^2,3^. Understanding the mechanisms that influence the turnover and stabilization of organic matter within soils is therefore critical for maintaining the proper soil structure formation, soil fertility improvement, and effective food production^4^.

Various studies highlighted the benefits of using different organic materials to improve the level of SOM and nutrients in soils^5–7^ as well as soil structure and mechanical properties^8^. However, the problem of how the digestate affects soil structural and mechanical properties, especially concerning soil aggregates, has been explored rarely.

Digestate, a by-product of anaerobic biomass fermentation in agricultural biogas plants, seems to be a promising waste material in this field^9^. Large subsidies from EU governments for biogas installations^10^ caused that by the end of 2018, more than 18,000 biogas plants had been registered, an increase of 192% compared to 2009 ^11^. Renewable energy produced in anaerobic digestion from organic waste from households, food and beverage processing, agriculture, and sewage treatment plants is a real advantage approaching the EU target that by 2020, 20% of all energy consumption should come from renewable sources^12^. However, the European Union produces approximately 180 million tons of anaerobic digestate annually^13^. Such large amounts of waste create a disposal problem and an urgent need for systemic solutions to effective biomass utilization. Organic waste application derived from post-production processes as a soil structural conditioner may decrease storage and disposal costs^14^.

The use of digestate to improve soil quality is in line with current European policy promoting green deal and the Circular Economy Action Plan, implemented by the European Commission in 2015, which encourages the reuse of by-products or waste to create further value at the end of a product’s life. The digestate reveals high potential as an effective soil fertilizer due to high organic carbon level^15^ and content of macro- and microelements well-available to plants^16,17^. So far, research has focused primarily on the agricultural benefits of using digestate for improving the chemical and microbiological properties of soil, as well as on environmental risks associated with using non-fully stabilized waste^18–20^. Several studies have examined the use of digestate as nitrogen fertilizer, considering nitrogen turnover and its partitioning pathways^17,21^. However, there is still a lack of knowledge about the impact of digestate on soil physical properties, such as pore size distribution, pore connectivity, and aggregate mechanical properties, all of which are directly related to proper soil structure formation^17,20,22–24^. In fact, deterioration of soil porosity and mechanical properties can lead to soil degradation by negatively affecting macro- and micro-scale processes: water movement and retention, root growth, nutrient uptake, soil vulnerability to crusting, compaction, wind and water erosion, aeration, temperature fluctuations, surface runoff, susceptibility to desertification, long-term organic carbon storage and availability in soil^25–32^. The scale of the problem is large because approximately 24% of the world’s land area is covered by degrading soils, which equals 35 million square kilometers or 3500 million hectares^33^. In turn, according to Montanarella and Panagos^34^, achieving land degradation neutrality by 2030 should be a prerequisite for subsequently attaining a climate-neutral continent by 2050.

It seems that digestate, a material rich in organic components, could significantly influence the soil’s mechanical properties and structure^22,24^. Sparse research shows that the effect may be difficult to predict due to the variety of digestate composition and application methods, soil type, and bioclimatic conditions, highlighting the need for comprehensive model research on the specific role of digestate in soil structure formation. Understanding how soil physical properties respond to digestate addition is crucial for overall soil performance and quality improvement.

The main objectives of this study was: (a) to determine the impact of digestate on the structural and mechanical properties of soils, (b) to determine the effect of soil properties like for example grain size composition, pH, and organic matter content, (c) to assess the potential of digestate to restore the quality of degraded soils. The experiment was based on model aggregates prepared by mixing different proportions of digestate (0, 1, 3, 5 10 an 15% (w/w)) with various soils. The main idea of such studies was to exclude the potential effect of environmental factors and soil heterogeneity, thus allowing us to find more clearer relationships between the organic amendment dose and the structure parameters of soil aggregates. Scanning electron microscopy (SEM), nitrogen adsorption, mercury porosimetry, bulk density measurements, and uniaxial compression tests were performed.

## Materials and methods

### Materials

Samples of five different soils were collected in the Lublin province (southeastern part of Poland) from 0 to 25 cm depth, then air-dried and passed through a 1 mm sieve. The classification, sampling location, and abbreviation of the studied soils were as follows:

- A: Abruptic Luvisol (degraded Podzolic soil from weakly loamy sand, 50°15′ N, 23°27′ E near Basznia village),
- B: Brunic Arenosol (degraded Podzolic soil from silt clay loam, 50°39′ N/22°65′ E near Biszcza village),
- C: Haplic Cambisol (Brown soil, 51°23’N/22°35’E near Zalesie village),
- D: Haplic Chernozem (degraded Chernozem, 50°54’N/24°02’E near Oszczów village),
- E: Haplic Fluvisol (Alluvial soil, 51°16’N/22°99’E near Dorohucza village),
- F: Stagnic Luvisol (Grey-brown soils, 50°81’N/22°69’E near Huta Turobińska village).

Moreover, soils A, B and D were also classified as degraded. In soil A chemical degradation occurred due to sulfur extraction, while in soil B - due to inappropriate cultivation and fertilization. Degradation of soil D was a result of reduced pH. Soils C, E, and F, covered by grasses and trees, were not degraded, and their structure has been changed over the years only by natural processes.

Post-fermentation sludge (PS) coming from an agricultural biogas plant after dry fermentation of corn (main component), rye, apple pomace, and distillery decoction, lyophilized and screened through 1 mm sieve was added to the soils in amounts of 0, 1, 3, 5 10 an 15% (w/w).

### Analysis of the physical and chemical properties of soil and digestate

The following properties were determined:

- granulometric composition by aerometric method^35^;
- pH in H_2_O and KCl (CX-505 digital pH-meter, Elmetron)^35^;
- total carbon (TC) (TOC MULTI N/C 2000, HT 1300 analyzer, Analytic Jena)^36^;
- organic carbon (C_org_) content by dichromate oxidation^37^;
- the ash content (heating the sample at 550 °C, 6 h, muffle furnace FCF 12 SP, Czylok)^38^;
- cation exchange capacity (CEC) determined by Mocek et al. ^35^ method;
- particle density (SPD) of the solid phase (Helium Ultrapycnometer 1000, Quantachrome)^39^;
- plant-available macroelements: phosphorus (UV-Vis spectrophotometry, SPECORD 50 PLUS, Analytik Jena), potassium, magnesium, and calcium (atomic absorption spectrometry, AAS contra AA 300, Analytic Jena)^40^;
- total zinc, lead, chromium, cadmium and copper content (extraction using acid digestion with HNO_3_-H_2_O_2_-HCl solutions, atomic absorption spectrometry, AAS contra AA 300, Analytic Jena)^41^.

### Preparation of model soil aggregates

Carefully homogenized distilled water-saturated pastes were prepared from the digestate and the soils in different proportions (1, 3, 5, 10, and 15% (w/w, dry mass) of the digestate content). The pastes were pushed into 1 cm diameter boreholes of 1 cm height Plexiglas plates and then air-dried. The excess material that sticks out from the forms was removed with fine sandpaper to create plane surfaces. Next, aggregates were removed from the boreholes and conditioned in a laboratory atmosphere (60% relative humidity and 20 °C) until constant mass. Details concerning model aggregate preparation can be found in other works^42–44^. The aggregates were abbreviated using the convention: soil + digestate (P) percentage (e.g., D + P 1%). The letters denoting particular soils are the sme as in paragraph 2.1. of the Materials section.

### Analysis of soil aggregates

Magnified images of the broken aggregates were taken using the Phenom ProX desktop scanning electron microscope (Thermo Fisher Scientific, Waltham, MA, USA). The imaging was conducted on samples coated with an 8 nm gold layer (sputter coater CCU-010 LV, Safematic GmbH, Zizers, Switzerland) in BSE (backscattered electron) and SEM-EDS (scanning electron microscopy coupled with energy dispersive X-ray spectroscopy) modes. The BSE micrographs provided information on pore size and complexity, while EDS illustrated the location of organic molecules in soil aggregates. Representative images were selected from at least 10 scans.

The bulk density (BD) of the air-dry aggregates was estimated by dividing the aggregate mass (minus its water content) by the aggregate volume measured through compulsive immersion in mercury. The water content in the aggregates was determined by weighing after overnight heating at 105°C^44^. Measurements were performed in 10 replicates.

Mercury intrusion porosimetry (MIP) measurements were performed using an Autopore IV 9500 porosimeter (Micromeritics, Norcross, GA, USA) on dried aggregates samples (105 °C, 24 h). Pore volume versus pore radius curves in the pore range from 3 nm to 360 μm were measured. From the experimental curves, pore size distributions, total volume of pores, V_t_ (cm³/g), average pore diameter, D_av_ (µm), total pore area, S (cm^2^g^− 1^) and total porosity (P_c_) of the aggregates were calculated. Detailed information on the method and the calculations can be found in Jozefaciuk et al.^45^.

Uniaxial compression tests were performed using the Electronic testing machine LabTest 6.10.1 (LABORTECH s.r.o., Opava, Czech Republic). The aggregate was placed vertically and forced by a piston with a speed of 10 mm·min^− 1^. The force was estimated with an accuracy of ± 0.05 N. The average breakage curves showing the dependence between the compression stress, σ (MPa) (load divided by the aggregate cross-section area), and strain, ∆L/L (relative aggregate deformation, equal to piston displacement divided by the aggregate height), were determined from at least six curves that were most similar among ten replicates that allowed for the elimination of the structural artifacts. From the average curves, Young’s modulus (YM) and maximum stress causing aggregate destruction (σ_max_) were determined for all aggregates^43,44^.

The low-temperature nitrogen adsorption isotherms, showing the amount of adsorbed nitrogen against its relative pressure (p/p_0_), were measured. From the isotherms, the specific surface areas, SSA (m^2^g^- 1^), were calculated using the standard BET Equation^46^.

### Statistical analysis

The statistical analyses, including averages, standard deviations (SD), and Pearson’s correlation matrix, were performed using STATISTICA 13.3. software (TIBCO Software Inc., USA). The one-way analysis of variance (ANOVA) with post-hoc analysis (HSD Tukey test) was used. The tests were carried out separately for each soil variant, with digestate concentration as the differentiating factor. The Shapiro-Wilk test was used to verify data normality. The significance level was estimated at α = 0.05. The relationship between selected physicochemical variables of soils and soil aggregates was presented as a matrix table that includes strong (0.6–0.79) and very strong (> 0.80), positive (+) and negative (-) correlations.

## Results

### Properties of the research materials

The physicochemical properties of tested soils were shown in Table 1.

**Table 1:** The physicochemical properties of tested soils - average values (± standard deviation).

	pH (H_2_O)	pH (KCl)	TC (mg·g^− 1^)	C_org_ (%)	SPD (g·cm^− 3^)	BD (g·cm^− 3^)	SSA (m^2^·g^− 1^)	CEC (cmol·kg^− 1^)	D_av_ (nm)	S (m^2^·g^− 1^)	V_t_ (cm^3^·g^− 1^)	*P*_c_ (%)	Sand (%)	Silt (%)	Clay (%)
A	5.96 ± 0.19	4.91 ± 0.06	12.01 ± 0.53	0.53 ± 0.05	2.62 ± 0.002	1.68 ± 0.03	1.55 ± 0.08	8.30 ± 0.17	2080.65 ± 27.65	2.28 ± 0.31	0.21 ± 0.01	33.67 ± 1.34	43	51	6
B	6.31 ± 0.13	5.49 ± 0.03	11.28 ± 0.63	0.90 ± 0.03	2.63 ± 0.003	1.78 ± 0.02	0.47 ± 0.00	6.86 ± 0.09	8923.24 ± 108.03	0.95 ± 0.13	0.16 ± 0.00	28.06 ± 0.65	86	12.5	1.5
C	3.98 ± 0.08	3.44 ± 0.05	32.60 ± 0.83	3.05 ± 0.01	2.42 ± 0.002	1.61 ± 0.02	0.27 ± 0.02	11.46 ± 0.49	9130.44 ± 61.08	1.22 ± 0.65	0.19 ± 0.00	31.68 ± 0.21	81	17	2
D	4.48 ± 0.02	3.60 ± 0.02	16.67 ± 1.63	1.33 ± 0.02	2.59 ± 0.003	1.67 ± 0.03	6.78 ± 0.67	17.05 ± 0.12	1321.69 ± 14.21	5.23 ± 0.11	0.22 ± 0.00	35.11 ± 0.15	5	80	15
E	5.96 ± 0.02	5.12 ± 0.03	15.69 ± 1.15	1.47 ± 0.02	2.47 ± 0.164	1.86 ± 0.01	4.29 ± 1.22	14.47 ± 0.11	5784.49 ± 209.23	3.76 ± 0.53	0.13 ± 0.00	24.00 ± 0.42	74	19	7
F	5.30 ± 0.23	4.43 ± 0.02	28.99 ± 1.69	2.49 ± 0.06	2.53 ± 0.002	1.50 ± 0.03	2.06 ± 0.48	16.09 ± 0.02	1732.67 ± 108.95	3.68 ± 0.64	0.27 ± 0.00	39.20 ± 0.12	15	71.5	13.5

A, B, C, D, E and F – soils used in the experiments; pH – the soil pH measured in water (H_2_O) or potassium chloride solution (KCl); TC – total carbon; C_org_ – organic carbon; SPD – solid phase density (particle density); BD – bulk density; SSA – specific surface area measured by nitrogen adsorption; CEC – cation exchange capacity; D_av_ – average pore diameter measured by mercury intrusion; S – the total area of pores measured by mercury intrusion; V_t_ – the total volume of pores measured by mercury intrusion, P_c_ – total porosity.

The contribution of sand fraction was the highest in B (Brunic Arenosol), C (Haplic Cambisol), and E (Haplic Fluvisol) soils, while silt dominated in A (Abruptic Luvisol), D (Haplic Chernozem), and F (Stagnic Luvisol) soils. Soils D and F contained more than 13% of the clay fraction.

The BD of studied soils ranged from 1.50 to 1.86 g·cm^- 3^, while SPD differed slightly from 2.42 to 2.63 g·cm^- 3^. It was in line with general BD values for mineral soils which range typically from 1.0 to 1.6 g·cm^- 3^ for clayey soils and from 1.2 to 1.8 g·cm^- 3^ for sandy soils. The critical BD values can start from 1.3 g·cm^- 3^, depending on the soil texture, above which the adverse processes related to plant root growth reduction can occur. Soils A and B contained relatively small amounts of TC and C_org_, indicating a deficiency of organic matter. The highest content was observed for soils C and F, for which C_org_ was 3.05 and 2.49%, and TC was 32.60 and 28.99 mg·g^- 1^, respectively. The soils were strongly to slightly acidic. The pH measured in H_2_O ranged from 3.98 to 6.31 and in the KCl solution from 3.44 to 5.49 pH units. Soil CEC decreased in the order D > F > E > C > A > B and was higher for soils containing more clay fraction (soil D and F) and organic carbon (soil from C to F). The highest D_av_ values were found in soils B and C, while several times smaller diameters were observed for soils A, F, and D. The latter soils were also characterized by a higher V_t_. The values of this parameter ranged from 0.13 to 0.27 cm^3^·g^- 1^ and were the lowest for soil E. All analyzed soils had SSA and S values that did not exceed 7 m^2^·g^- 1^.

Table 2 presents the main physicochemical properties of the digestate. The lyophilized material was dark green to black with visible undecomposed plant fragments used for the fermentation. The digestate was alkaline (pH in H_2_O and 1 M KCl: 8.58 and 5.11, respectively). The SSA value was relatively small and reached 4.43 m^2^·g^- 1^. The D_av_ did not exceed 1805 nm, with the V_t_ − 0.29 cm^3^·g^- 1^. The BD and SPD values were considerably lower than for soils and reached 1.00 and 1.55 g·cm^- 3^, respectively. Values of BD reported by various sources as between 0.30 g cm^– 3^ to 1.24 g cm^– 3^ are typical for this kind of material and depend on digestate feedstock materials and the technology adopted.

The relatively low ash content (34.5%) indicated a significant content of organic matter. This was confirmed by the high C_org_ (28.94 mg·g^- 1^) and the TC (343.27 mg·g^- 1^) concentrations. The digestate contained a significant amount of available macroelements such as P, Ca and Mg (see Table S1 in supplements). The P concentration was slightly more than 5790 mg·kg^- 1^, Ca − 4463 mg·kg^- 1^, and Mg − 4750 mg·kg^- 1^. The total content of heavy metals (see Table S1 in supplements) did not exceed standards for using organic sludge for agricultural purposes following the Polish Regulations of the Minister of the Environment on the use of municipal sewage sludge. The content of Cu was 37.53 mg·kg^- 1^, Zn − 480.61 mg·kg^- 1^, Pb − 12.95 mg·kg^- 1^, Cd – 2.47 mg·kg^- 1^ while Cr was not detectable, which indicated the possibility of safe use of the above material in soils. Moreover, no live eggs of parasites such as Ascaris sp., Trichuris sp., Toxocara sp., or Salmonella bacteria were found.

**Table 2:** Physicochemical properties of digestate (P) - average values + standard deviations.

	pH (H_2_O)	pH (KCl)	TC (mg·g^− 1^)	C_org_ (%)	Ash (%)	SPD (g·cm^− 3^)	BD (g·cm^− 3^)	SSA (m^2^·g^− 1^)	D_av_ (nm)	S (m^2^·g^− 1^)	V_t_ (cm^3^·g^− 1^)	*P*_c_ (%)
**P**	8.58	5.11	343.27	28.94	34.50	1.553	0.996	4.43	1804.8	10.33	0.29	29.4
**SD**	0.01	0.01	8.95	0.78	0.38	0.005	0.021	0.62	135.09	0.69	0.02	1.33

P – digestate used in the experiments; pH – the soil pH measured in water (H_2_O) or potassium chloride solution (KCl); TC – total carbon; C_org_ – organic carbon; SPD – solid phase density (particle density); BD – bulk density; SSA – specific surface area measured by nitrogen adsorption; CEC – cation exchange capacity; D_av_ – average pore diameter measured by mercury intrusion; S – the total area of pores measured by mercury intrusion; V_t_ – the total volume of pores measured by mercury intrusion, P_c_ – total porosity.

**Fig. 1:**
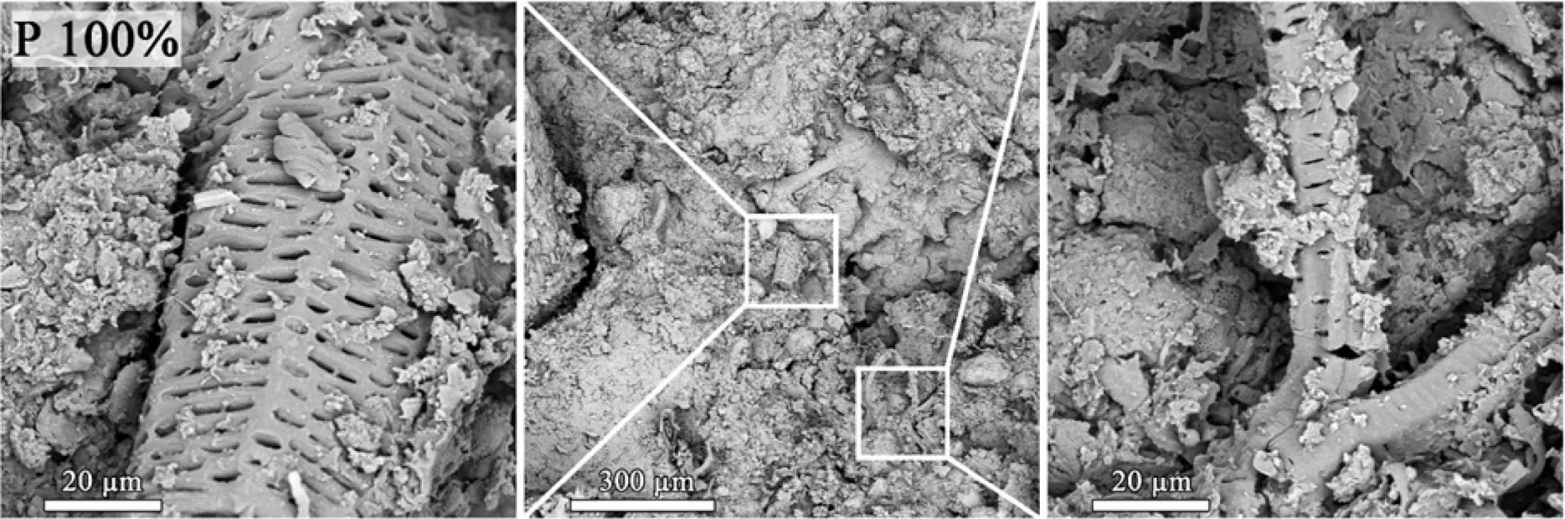
Representative SEM images of the surfaces of post-fermentation sludge. While the images on the left and right show structure examples in higher magnification (5000x), an image in the center shows an overall view of digestate (250x).

The SEM images showing the morphological features of the digestate presented in Fig. 1 revealed the structures of plant residues affected by anaerobic fermentation and solid mineral particles grouped in larger agglomerates that occur separately or close to plant particles. The material contained irregular ridges and channels, fibers, and pores originating from the vascular tissue of plants. The digestate surface was non-uniform and porous with pits, incrustations, and scattered roughness.

### Structural properties of the model soil aggregates enriched with digestate

**Fig. 2:**
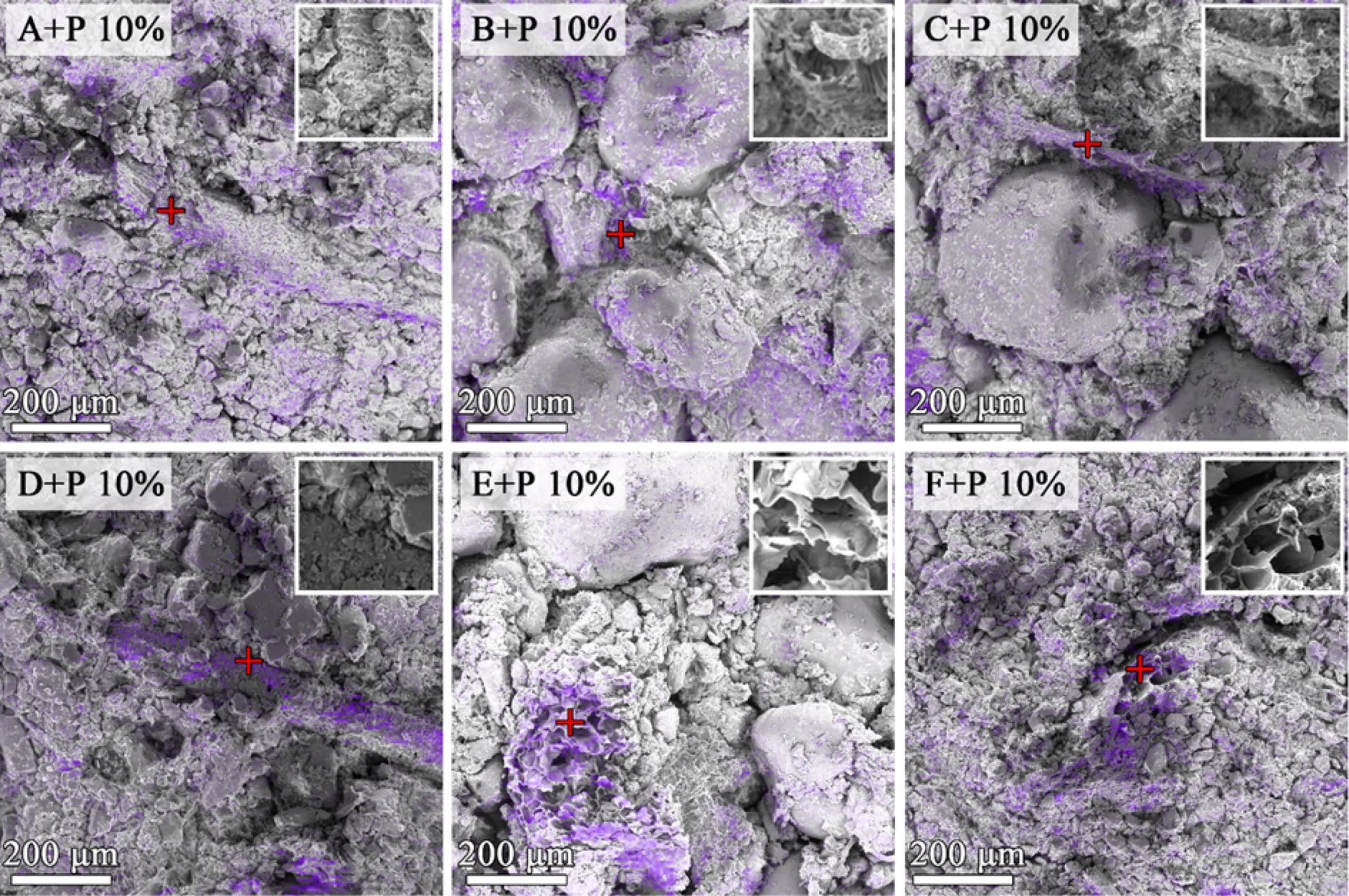
Representative SEM-EDS images of the surfaces of broken soil aggregates containing 10% of the digestate. The location of carbon is marked in violet. Higher magnifications (10 × 10 μm images) of the areas with red plus signs are presented in the upper right corner.

Examples of SEM/EDS images of the aggregates enriched with 10% of the digestate are presented in Fig. 2. From these images, the microstructure and the location of organic particles are visualized.

Larger digestate particles or conglomerates were mostly surrounded by soil mineral particles (see soils D and F). The smaller ones were located inside interparticle pores or on mineral particle surfaces (see soils B and C).

Porosity is the best indicator of soil structure quality. Size, continuity, and arrangement of pores in soil matrix define the complexity of soil structure and may help understand its modifications induced by digestate.

**Fig. 3:**
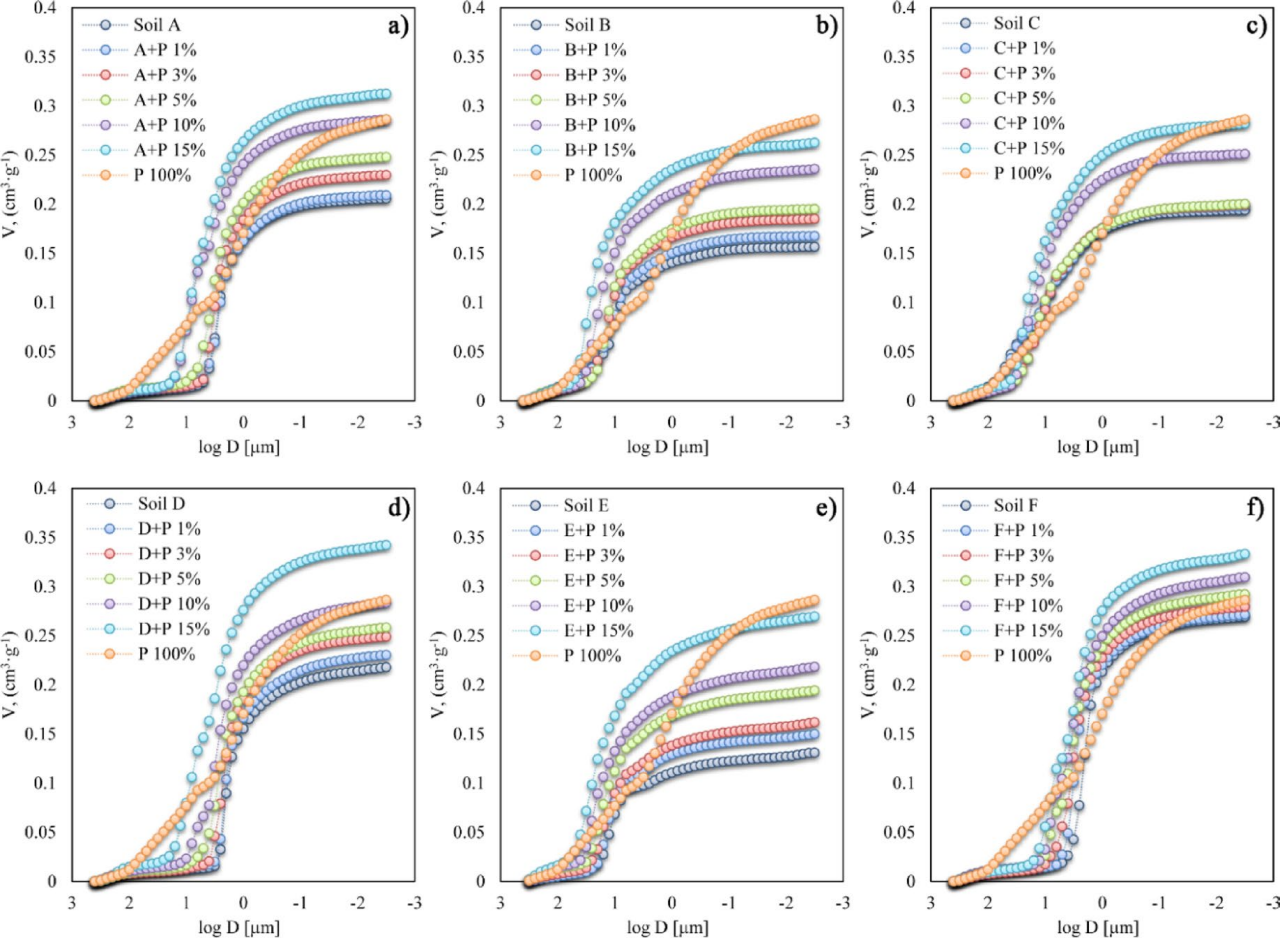
The cumulative curves for the tested soil aggregates containing various proportions of digestate (**a**–**f**). Particular plots show the tested soil (A-F). For better comparison, the curve for pure organic additive (P 100%) is drawn in each plot. The bars with experimental deviation were not included to reach the figure transparency.

Figure 3. shows the relationships between the intruded mercury volume and pore diameter for the tested aggregates. All curves were close to sigmoidal, indicating that the pore volume increased with the digestate concentration. The highest volume was observed for the aggregates of the control soil D and F, while the lowest one for soil E. For the control soils, a rapid increase in the pore volume starts at around 5 μm pore diameter for soils A, D, and F, from around 35 μm pore diameter for soils B and E, and around 55 μm pore diameter for soil C. The rapid mercury intrusion for pure digestate (P 100%) was observed around 140 μm pore diameter.

**Fig. 4:**
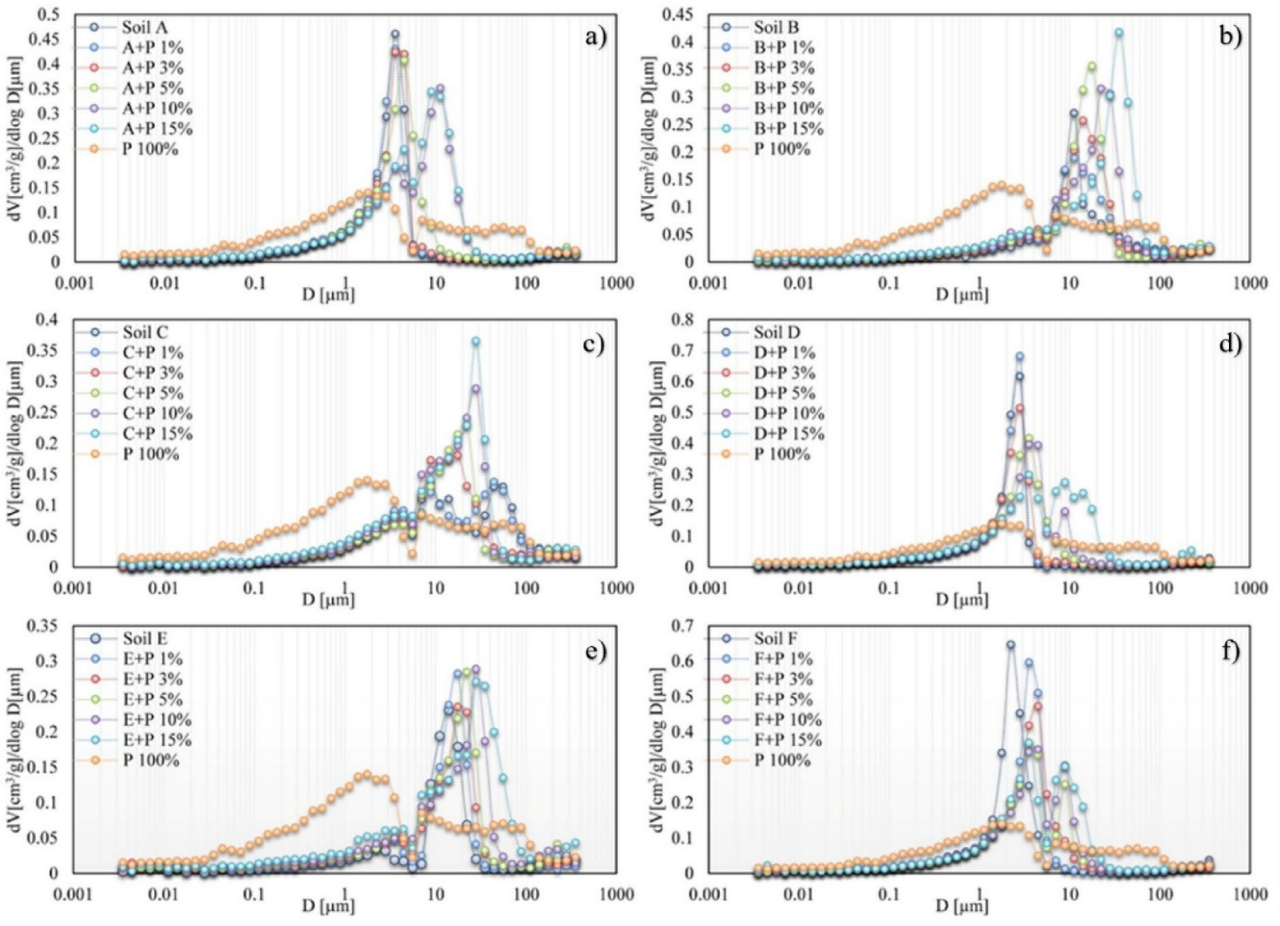
Pore size distribution curves for soil aggregates containing various proportions of the digestate (**a**–**f**). The control soils and the pure digestate (P 100%) are included for easier comparison. Note different scales of vertical axis for various groups of aggregates. The bars showing experimental deviations were not included to reach figure transparency.

The pore size distribution curves of soil aggregates are presented in Fig. 4. These curves are unimodal or bimodal depending on soil type and digestate concentration. The pore size distribution of the pure digestate is the broadest. Usually, increasing the digestate concentration in soil aggregates shifted the main peak toward pores of a larger diameter. The exception was soil C, for which the main peak of the aggregate’s pore size distribution moved towards a smaller diameter of pores with digestate concentrations up to 3%. From that point, higher digestate doses started to increase pore size diameter.

**Fig. 5:**
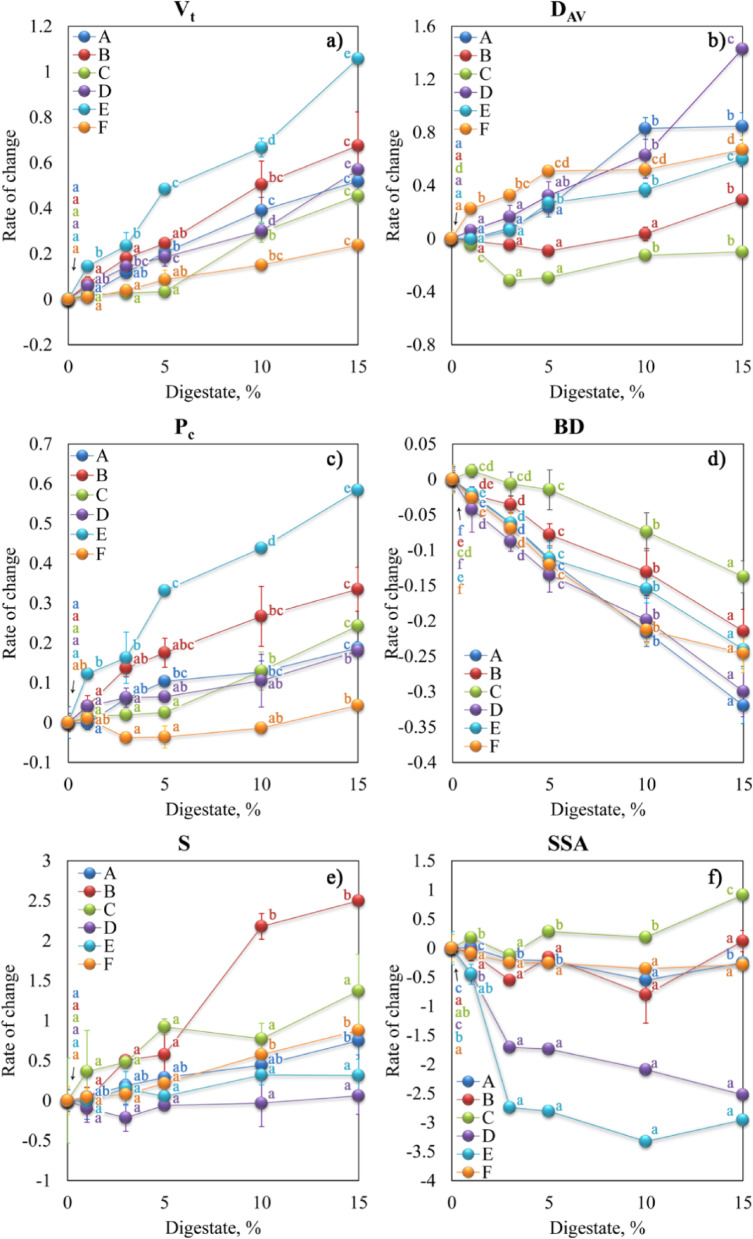
Rate of changes in: (**a**) total pore volume (V_t_), (**b**) average pore diameter (D_av_), (**c**) total porosity (P_c_), (**d**) bulk density (BD), (**e**) surface area of pores (S), and (**f**) specific surface area (SSA) with increasing digestate concentration.

Dependencies of mercury intrusion parameters (P_c_, V_t_, S, and D_av_), BD, and SSA on the digestate concentration are presented in Fig. 5. The figure shows how many times the given parameter estimated in the digestate amended soil (X_D_) differs from its value in the control soil (X_0_), according to Eq. (1):

1 $${\text{Rate of change }}={\text{ }}\left[ {\left( {{{\text{X}}_{\text{D}}}} \right){\text{ }} - {\text{ }}\left( {{{\text{X}}_0}} \right)} \right]{\text{ }}/{\text{ }}\left( {{{\text{X}}_0}} \right){\text{ }}={\text{ }}\left( {{{\text{X}}_{\text{D}}}} \right){\text{ }}/{\text{ }}\left( {{{\text{X}}_0}} \right){\text{ }} - {\text{1}}$$

Note that the above approach gives positive values for a parameter increase and negative values for its decrease after the sludge addition.

Increasing digestate concentration in aggregates increased the V_t_ and decreased the aggregate BD. The greatest changes in V_t_ were observed for soil B and E, while the minor ones characterized aggregates of soil F. The smallest changes in BD were observed for soil C, especially when doses of digestate were not greater than 5%. In turn, the rate of BD changes for aggregates of soil F decreased for digestate doses of 10 and 15%.

The P_c_, S, and D_av_ parameters usually increased with digestate concentration. The smallest changes of P_c_ were observed for soil F and of S for soil D. In turn, higher digestate concentrations (10 and 15%) strongly modified the surface area of pores in aggregates of soil B. The SSA diminished strongly for soils D and E, while for the other soils, the rate of SSA change was small and did not always show a clear trend. The D_av_ values increased rapidly for aggregates of soil D, A, F, and E and in the case of higher doses of digestate for soil B. The digestate addition decreased the average pore diameter, D_av_, of soil C.

The statistical significance of the differences between means was estimated at the level of α = 0.05 and presented in supplementary materials (see Table S2).

### Mechanical properties of the soil aggregates enriched with digestate

The parameters of the soil strength refer to the inherent ability of soil to resist the disruptive forces that cause fracture or rupture of the soil. Addition of digestate may contribute to microstructural development and soil resilience against external forces as changes in soil mechanical strength strongly depend on soil porosity, interparticle bonds, clay content and mineralogy, organic carbon content, and surface characteristics.

**Fig. 6:**
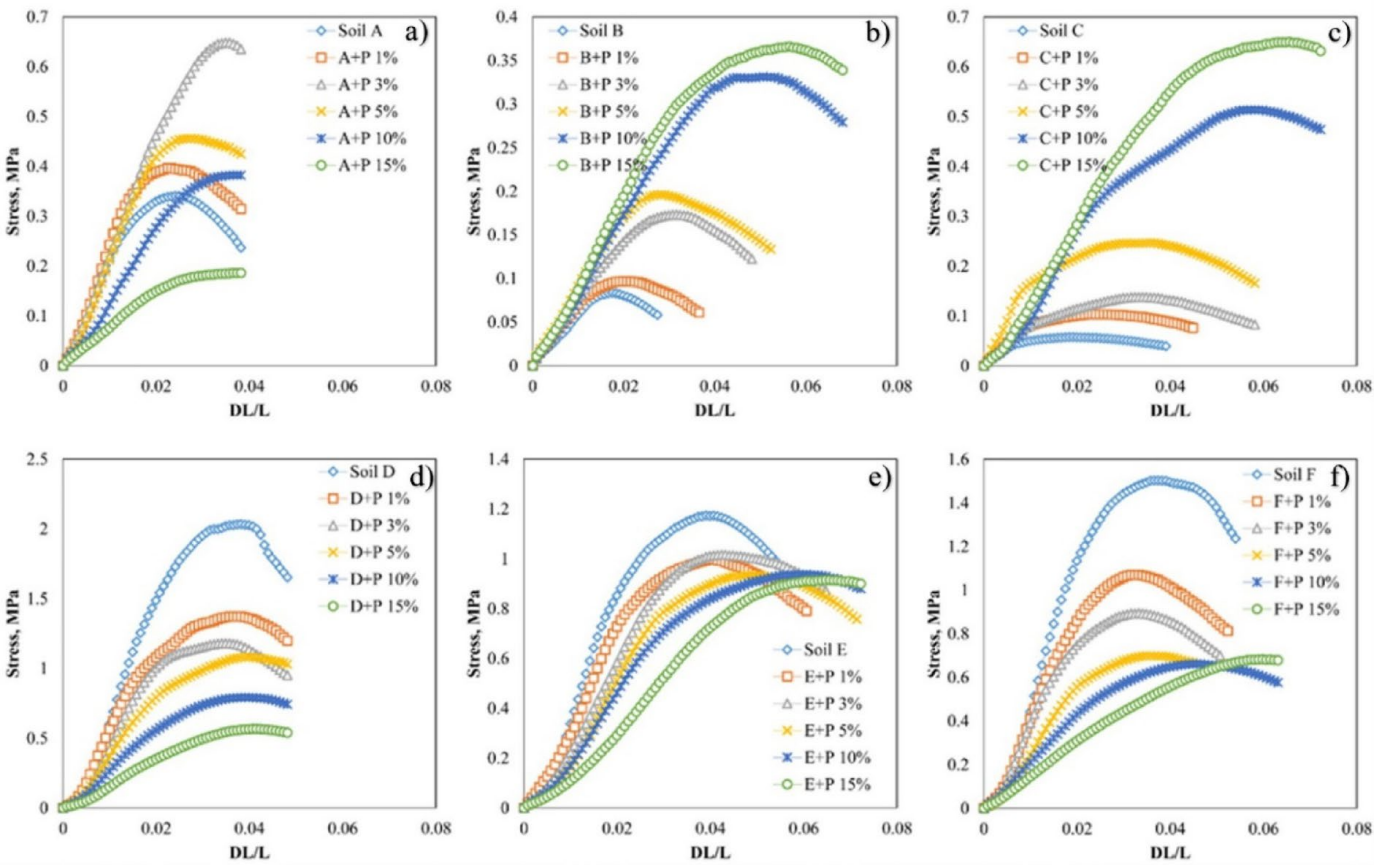
Exemplary stress-strain curves for the soil silt aggregates amended with various proportions of digestate (**a**–**f**). Selected curves represent the average. Note different scales on the vertical axis for various aggregate groups. The bars with experimental deviation were not included to reach figure transparency.

The results of the average stress-strain curves from uniaxial compression tests are presented in Fig. 6.

The curves are characteristic for a ductile breakage mode i.e. significant plastic deformation prior to failure, occurring at uneven and distributed cracks^47^. The model aggregates from pure digestate exhibited the highest mechanical strength, even several times higher than soils or soil-digestate mixtures. Soils D, F, and E without organic additive had the highest σ_max_, and increasing digestate concentration decreased the mechanical strength of the aggregates. The opposite trend was observed for aggregates of soils C and B, for which digestate significantly increased aggregate strength more than tenfold and fourfold, respectively. In the case of soil A, the mechanical strength increased but only up to 3% of organic additive and then decreased with digestate concentration.

**Fig. 7:**
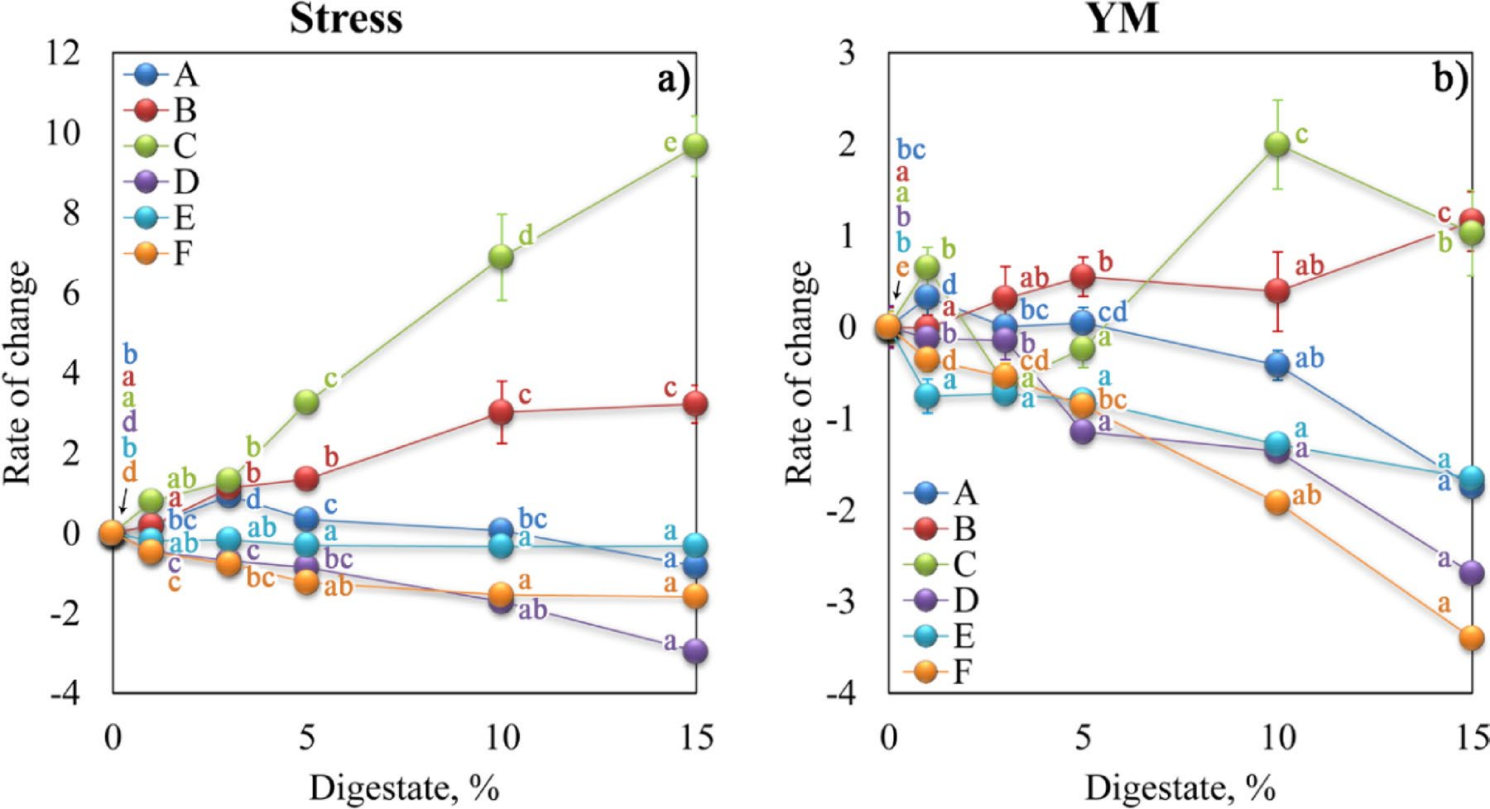
Dependencies of: (**a**) the maximum stress (Stress) and (**b**) Young’s modulus (YM) of the studied aggregates on the digestate concentration.

Analogically to Fig. 5, Fig. 7. presents the rate of changes in the σ_max_ and YM for aggregates with various digestate concentrations, respectively. The statistical significance of the differences between means was shown in supplementary materials (see Table S2).

Soil C, B, and D revealed the greatest rate of changes in aggregate strength, with σ_max_ increase for soil C and B and a maximum stress decrease for soil D. A similar rate of change was observed for soils A and E as well as F and D. In the case of YM, the highest increase revealed aggregates of soils C and B with digestate concentration of 15%. In turn, a significant decrease occurred for aggregates of soils F and D. Except for aggregates of soils C and B, digestate decreased the values of YM of soil aggregates.

### Correlation analysis of the obtained results

Table 3 presents the Pearson correlation matrix for selected variables. In this table, dark grey and light grey colors mean very strong (> 0.80) and strong (0.60–0.79) correlations, respectively. The “+” sign means a positive correlation, while the “-” sign means a negative one.

**Table 3:**
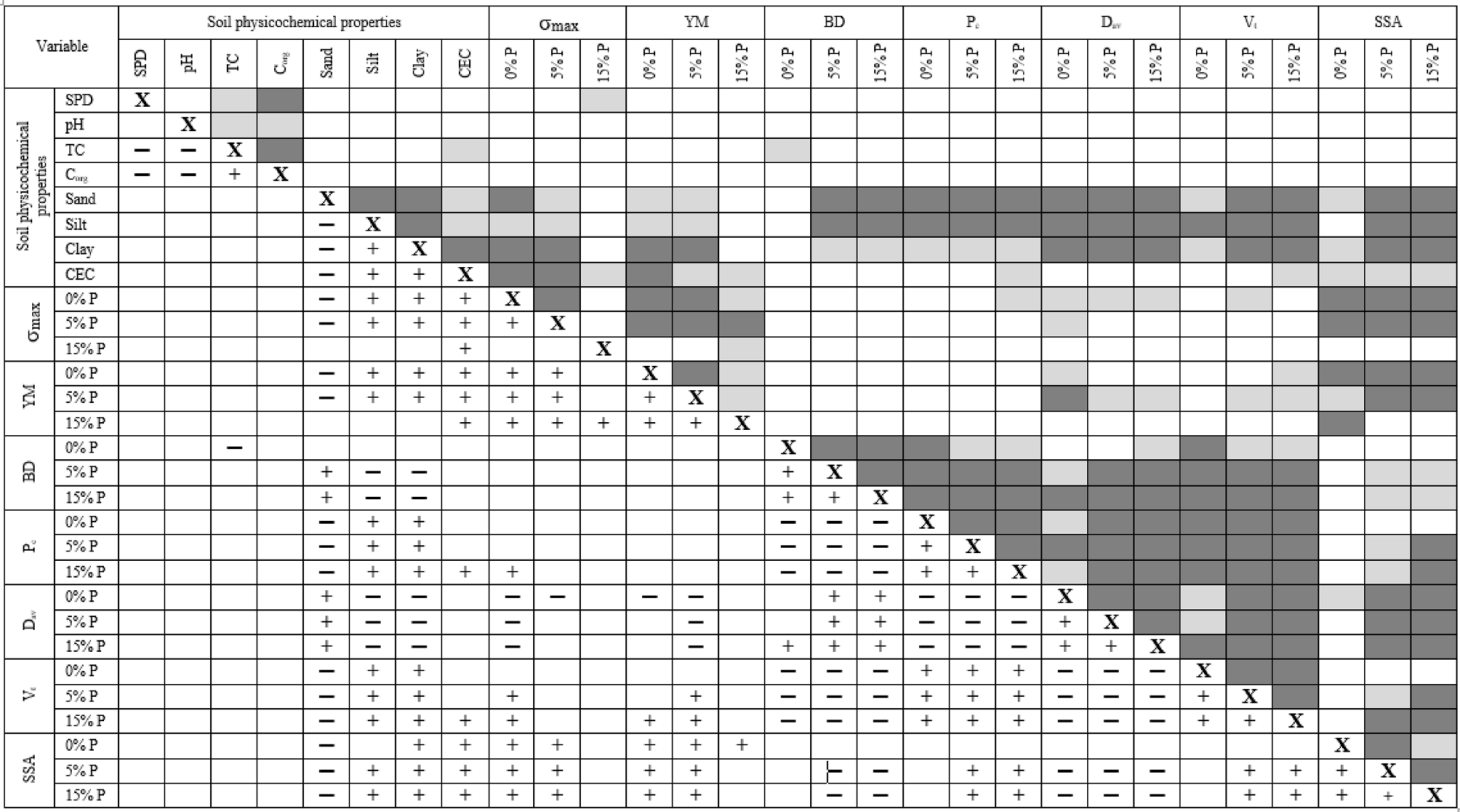
Pearson correlation matrix for selected variables. Dark grey and light grey colors mean very strong (>0.80) and strong (0.60-0.79) correlations.

P – digestate; pH – pH measured in water (H_2_O); TC – total carbon; C_org_ – organic carbon; SPD – solid phase density; BD – bulk density; SSA – specific surface area; CEC – cation exchange capacity; D_av_ – average pore diameter; V_t_ – the total volume of pores, P_c_ – total porosity; σ_max_ – maximum stress at aggregate breakage.

The granulometric composition of the soil was an important factor in determining the structural properties and mechanical strength of model soil aggregates. A strong and/or very strong negative correlation between the sand fraction versus P_c_, V_t_, SSA, σ_max_ (≤ 5% P), and YM (≤ 5% P) was observed. As a consequence, a positive relationship was found between the silt and clay fractions and the above parameters. Soil granulometric fractions also correlated with other parameters studied. In general, BD and D_av_ correlated positively with sand fractions and negatively with silt and clay fractions. While σ_max_ (≤ 5% P) and Young’s modulus (≤ 5% P) showed a strong positive correlation with the SSA, negative relationships were found with D_av_. It is worth noting that there was no significant correlation at 15% digestate addition. The BD correlated positively with D_av_ and negatively with V_t_ and SSA, especially at higher doses of digestate. Significant positive relationships were also found between σ_max_, YM, and SSA versus soil CEC. In general, any substantial correlation between the SPD, pH, TC, or C_org_ with other tested parameters was not observed.

## Discussion

### Effect of the digestate on structural and mechanical properties of soils

The impact of digestate on the structural and mechanical properties of soils appears to be complex and dependent on many factors and soil parameters. While the seasonal variations in soil physical properties, such as soil bulk density, porosity, and aggregate stability, make these dependencies even more complicated and difficult to predict^24,48^, our study focused on model soil aggregates and aimed to uncover the underlying relationships governing the interactions between digestate and soil in the absence of external influences.

The BD and porosity are the main indicators of soil compaction. In our studies, the digestate significantly reduced the BD and increased the porosity of soil aggregates (Fig. 5d) that may be connected by its lower bulk density than that of the soils^18^. Similarly, Mayerová et al.^24^ found that digestate and compost fertilizers significantly decreased soil BD and increased porosity compared to the control and mineral fertilized treatment. Moreover, they observed more significant improvement in the physical properties of sandy soil amended with organic fertilizers than silty clays. This observation does not align with our results for Haplic Cambisol (C) (predominance of the sand fraction), where the smallest changes in the BD were observed, especially when doses of digestate were not greater than 5% (Fig. 5d). The observed effect was probably related to digestate particles that changed pore size and their tortuosity. Digestate particles finer than sand particles filled the interparticle pores, resulting in a decrease in average pore diameter and more effective packing in soil matrix (Figs. 2 and 5b). In the case of Haplic Chernozem (D) and Abruptic Luvisol (A), which were characterized by high silt fraction content, the increase in the digestate concentration caused the greatest change in the BD, suggesting the creation of additional spaces close to organic particles. The effect of organic fertilizers on changes in the BD and soil porosity was confirmed by Rivenshield and Bassuk^49^. They suggested that the application of organic conditioner on fine texture soil may not result in a large change in the physical properties and that greater amounts of organic fertilizers are needed to improve its physical properties. This is consistent with our results as the digestate doses higher than 5% caused more intense changes in all tested soils.

The results proved that digestate addition to soils caused significant changes in aggregates’ porosity, especially for higher digestate concentrations. The pore size distributions were shifted towards higher pore diameters (Fig. 4), and the average pore diameter, pore volume, and total porosity increased in almost all soils (Fig. 5). This finding is in line with Skic et al.^14^, who reported that digestate induced pore space reallocation with a shift from small pores to larger pores thus, promoted soil structural development. The above data were also supported by De Gryze et al.^50^, who found an increase in soil void porosity within the 27–67 μm diameter range after fresh organic residue application. They also noticed that pore creation could result from various mechanisms, including shrinking and swelling of soil and organic particles and decomposition of organic matter by microorganisms. Similar findings were reported by Dlapa et al.^51^, who observed that higher soil organic carbon content promotes the formation of elongated pores with a characteristic diameter of 15–27 μm. They also found that the creation of micro-cracks improved soil hydraulic properties due to the enhanced ability of structural pores to drain water during rainfall.

It should be mentioned that some studies revealed the deterioration of structural properties of soil after enrichment in organic matter. In line with Jaša et al.^52^, the application of digestate reduced the volume of storage and transmission pores, deteriorated aggregate stability, and increased soil compaction. As a result, important processes such as drainage, aeration, and plant growth could be reduced^14^. In our studies, this relationship was visible for Haplic Cambisol (C) aggregates. The digestate application resulted in a higher number of contact points between soil particles that temporarily diminished average pore diameter and increased soil aggregates’ mechanical strength^53^. The diminished air permeability and relative gas diffusivity for the soil with applied organic matter were observed by Kuncoro et al.^54^. According to their studies, organic matter could block soil pores and increase the capillary water in the pore necks. This explanation was partly supported by the results of Schneider et al.^55^, who showed that coarse organic matter fraction caused the occlusion of pores and increased the hydrophobicity of soil components, leading to diminished soil hydraulic conductivity.

The SSA characterizes the properties of the solid phase of soil and usually correlates with soil sorption capacity, ion exchange capacity, retention and transport of water, chemicals, and microorganisms, swelling, and dry-aggregate stability^56^. The addition or removal of organic matter modifies the external SSA of soil particles, which has been the subject of previously published studies^57,58^. According to Sikora et al.,^56^ changes in SSA caused by various organic additives may result from several interacting factors such as organic matter and ash quality, plant residue amount and type, farming practice, and soil type. In the case of our studies, the addition of digestate led to a decrease in SSA of Haplic Chernozem (D) and Haplic Fluvisol (E) that could result from fine pores occlusion, however, the trends were not clear for other soils (Fig. 5f). These changes were closely correlated with sand, silt, and clay fractions (Table 3). Compared to other soil components, the clay fraction possesses a large specific surface area, which makes it a very reactive soil constituent in physicochemical processes, including organic matter stabilization^59^.

Organic matter of digestate used in our studies affected aggregates’ mechanical strength, and these changes were related to the dose of the additive (Fig. 7). The size and direction of the effect depended on both the textural properties of the soil and the chemical compositions of the digestate. Abiven et al.^60^ found a strong correlation between aggregate stability and the fraction of organic product that decomposes. Greater stability of soil aggregates enriched with organic fertilizer was demonstrated previously by Mayerová et al.^24^ on sandy and clay soils, however, these results differed significantly depending on the digestate type. They attributed the increased aggregate stability to the increased content of total carbon and nitrogen and the content of water-extractable carbon. This seems to correspond well with our results because the digestate used in our studies contained a high amount of total and organic carbon.

The mechanical stability of soils should decrease with a decrease in bulk density and an increase in pore volume^61,62^. In our studies, the bulk density decreased, and the pore volume increased with the digestate addition for all soils (Fig. 5), therefore we expected that the mechanical stability would decrease for all soils with the digestate addition. However, two soils, Brunic Arenosol (B) and Haplic Cambisol (C), exhibited the opposite trend (Fig. 6b and c). For the latter soils, in contrast to the other ones, the pore diameter of the digestate enriched samples was generally smaller than that of the control soils (Fig. 5b). Based on the above observations, we can outline several possible mechanisms influencing the digestate effect on soil porosity and strength:

1. Smaller digestate particles are located within larger pores of the soil matrix, which leads to an increase in bulk density, a decrease in pore volume and radii, and an increase in soil strength. This mechanism seems preferred when the soil matrix contains pores larger than organic particles (sandy soils).
2. Larger digestate particles are located among soil grains, which creates additional porosity, increasing pore volume and radii, decreasing bulk density, and decreasing soil strength. This mechanism seems to be preferred when the soil matrix contains pores smaller than the size of organic particles (clayey soils).
3. Organic matter forms coatings on soil particles by adhesion or polyvalent cations bridging. In this mechanism, water soluble organic matter should play a dominant role. If the coatings do not overlap and the coated particles (larger than noncoated ones) occur separately, the pore volume and radii increase, the bulk density decreases, and so does the soil strength. The opposite situation arises if the coatings overlap: the pore volume and radii decrease, the bulk density and the soil strength increase.
4. Strong interactions between organic and inorganic particles may lead to soil strengthening regardless of porosity increase and a decrease in bulk density.

Stronger interactions between organic and inorganic particles than between inorganic particles themselves may explain an increase in the strength of soils B and C with the digestate addition. Of course, strong interactions between organic substances and inorganic particles are likely to occur in the other soils, as well, however, the other strength-governing mechanisms seem to dominate. Since the above mechanisms are likely to occur simultaneously, the soil behavior prediction under the digestate effect is extremely difficult.

### Environmental potential of digestate in the restoration of degraded soils

Knowledge of digestate behavior and fate in different soils may help to counteract unfavorable phenomena related to soil degradation. Three out of the six soils tested showed signs of degradation. Soil A from Basznia was chemically degraded due to contamination from a nearby sulfur mine. Soil B from Biszcza was degraded due to improper cultivation and fertilization. Soil D from Oszczów was degraded due to a reduced pH level. Positive effects on porosity and pore size distribution characteristics (which increased with the concentration of the additive), bulk density (which decreased with the concentration of the additive), and specific surface area (which increased for A and B and decreased for D with the concentration of the additive) were observed. The results showed that digestate improved soils quality by increasing organic matter content and soil structure stabilization. The creation of structural porosity may favor water retention and a high rate of rainfall infiltration to soils and reduce soil susceptibility to surface runoff and accompanying erosion. Finally, optimal rates of digestate can improve physical soil properties, promoting plant growth and microbial activity.

When determining the effect of post-fermentation sludge on the mechanical strength of soils, we found that the granulometric composition was crucial. Coarse particles dominated for chemically and physically degraded soils (soil A and B, respectively), while for soil D, classified as chemically degraded due to the decrease in pH level, a decrease in strength was noted with the increase in the concentration of post-fermentation sludge. It seems that organic matter improves soil structure by bonding loose soil particles into strong aggregates in sandy soils. On the other hand, the organic material loosens the structure of clayey soils that often have high tensile strength values^63^. Changes in tensile strength influence soil tillability, seedling emergence, root growth, and other soil processes^64^, therefore our observations have important implications for soil management practices to maintain proper soil structure or avoid over-compaction problems in crop production.

The potential use of biodegradable waste to enhance soil quality and productivity while complying with regulations, standards, and norms is a focal point of many research activities^65^. Many available studies showed that organic waste can partially replace traditional mineral and organic fertilizers, resulting in improvement of soil organic carbon, nutrient and water level, electrical conductivity, and soil microflora of degraded soils^66–70^. Our results demonstrate that digestate can provide several other benefits, including aggregate formation, improved aeration, porosity, and structure stability. The low or undetectable amount of heavy metals in the studied digestate means that the use of this particular material for remediation purposes would not pose a risk to the subsoil and groundwater secondary contamination^71^. Together with the fact that digestate belongs to an inexpensive source of organic matter^72^, it should be considered as a prospective material for restoration of physically eroded soil quality.

The production and management of digestate are subject to several regulations and rules concerning various aspects of digestate production, quality standards, application rates, and environmental considerations. Individual member states of the European Union generally have specific regulations and guidelines for digestate management to ensure compliance with EU directives and to address local environmental and agricultural needs. In general, using digestate for environmental purposes requires its chemical and bacteriological safety. Regardless of the purpose of use (improving chemical, physical, structural, or mechanical properties of soil), the total content of heavy metals of digestate should be determined and controlled due to the high dynamics of organic matter biodegradation and the potential threat of heavy metals mobilization. The content of heavy metals should meet the criteria specified in the national regulations of the relevant ministry. Knowledge about pH and organic carbon content of the digestate also seems important because materials of organic origin, apart from their structure-forming properties, could deteriorate the quality and functions of the native soil. Since our results suggest that different types of soils respond differently to the addition of digestate, to improve the mechanical and structural properties of soils, the optimal dose of digestate should be determined based on the soil’s physical characteristics. The crucial information may be the evaluation of bulk density, pore volume, and mechanical strength of a given soil. These parameters allow for the assessment of soil degradation in the three most important physical aspects. Additionally, knowledge about texture could be an important hint of how the soil will react to digestate addition.

The question about the optimal dose of digestate is complex and should be considered individually for specific soil and the properties of organic material obtained by the anaerobic digestion process. In our studies, a wide range of digestate concentrations was checked to show that depending on the type and needs of a given soil, even lower doses e.g. 1–5%, could significantly modify soils’ structural properties. Therefore, applying lower doses or doses divided over a longer time could be a great advantage when maintaining chemical and biological safety and counteracting unfavorable phenomena such as excessive accumulation of elements and increasing pH or salinity. The rational use of digestate should be a compromise between a noticeable effect of soil structure improvement and the lowest possible dose of this conditioner.

## Conclusions

The development of society is closely linked to the need for increased crop yields. This demand depends on improving soil fertility and restoring the proper structure of degraded soils. The significant emphasis on eco-friendly solutions suggests the application of waste materials like digestate. Our research aimed to assess the impact of digestate on soil structural and mechanical properties, eliminate the effect of external environmental factors, and provide a foundation for future research. Due to degradation becoming an increasingly common problem that endangers agricultural soil fertility and productivity, the impact of organic additive on degraded soils was also examined. The research led to the following significant conclusions:

1. The digestate contained fine structures and plant residues with macro- and micropores. A high total and organic carbon content in digestate made it a valuable material for improving soil structural and mechanical properties.
2. The addition of digestate, in most cases, increased the total porosity, average pore diameter, and total pore volume while decreasing aggregates’ bulk density. Digestate reduced the specific surface area of the soil with a high content of sand fraction (E) and degraded chernozem with a high content of clay fraction (D), however, the changes were not consistent in the case of other soils. Except for sandy soil (C), increasing digestate concentration shifted the pore size distribution toward larger diameters. The above changes were closely related to the soil granulometric composition. Furthermore, the dominance of the sand fraction was associated with faster and more intense changes in the parameters’ values. The porosity and specific surface area of degraded soils responded differently to the addition of digestate, which emphasizes the need to adjust the waste dose to a specific type of soil.
3. The mechanical strength parameters were closely correlated with soil grain size composition. The digestate reduced the strength of soils with high initial strength (soils with a predominance of silt and clay fractions) and increased this parameter in soils with low initial stress required to destroy the aggregate (sandy soils). Our findings highlight the potential for using the digestate to enhance the stability of poorly aggregated soils.
4. The digestate addition can enhance soil structure and mechanical properties, thus eliminating or mitigating unfavorable changes in soil structure, such as compaction by vehicles and machines or inadequate tillage procedures. For satisfactory effects, proper rates of the conditioner based on soil textural properties and organic carbon content should be determined.

## Supplementary Information

Below is the link to the electronic supplementary material.

Supplementary Material 1

## Data Availability

Data sets generated during the current study are available from the corresponding author upon reasonable request.
