# Supplementary material for: Utilization of post-fermentation sludge as a soil structure and strength conditioner
Source: Sci Rep. 2025 Nov 6;15:38982. doi: 10.1038/s41598-025-22871-w (PMC12592455; doi:10.1038/s41598-025-22871-w)
Supplement: Supplementary file 1 — Supplementary Material 1 [file 41598_2025_22871_MOESM1_ESM.docx]

Supplementary Materials for

**Utilization of post-fermentation sludge as a soil structure and strength conditioner.**

Angelika Gryta^1^, Patrycja Boguta^1∗^, Grzegorz Józefaciuk^1^ & Kamil Skic^1^

^1^ Institute of Agrophysics, Polish Academy of Sciences, Doświadczalna 4 str., 20-290 Lublin, Poland

^∗^Corresponding author: p.boguta@ipan.lublin.pl

Table S1. The content of available macroelements and total heavy metals in the studied organic additive - average value (± standard deviation).

|  | **K_av_**  **(mg·kg^-1^)** | **Mg_av_**  **(mg·kg^-1^)** | **Ca_av_**  **(mg·kg^-1^)** | **P_av_**  **(mg·kg^-1^)** | **Cu**  **(mg·kg^-1^)** | **Pb**  **(****mg·kg^-1^)** | **Zn**  **(mg·kg^-1^)** | **Cd**  **(mg·kg^-1^)** | **Cr**  **(mg·kg^-1^)** |
| --- | --- | --- | --- | --- | --- | --- | --- | --- | --- |
| **P** | 38.95 | 4750.07 | 4463.47 | 5790.52 | 37.53 | 12.95 | 480.61 | 2.47 | n.d. |
| **SD** | 0.14 | 474.06 | 42.15 | 763.27 | 9.41 | 0.25 | 79.40 | 0.11 | n.d. |

Abbreviation: P – digestate used in the experiments; K_av_ - content of potassium available to plants; Mg_av_ - content of magnesium available to plants; Ca_av_- content of calcium available to plants; P_av_ - content of phosphorus available to plants; Cu - total content of copper; Pb – total content of lead; Zn – total content of zinc; Cd – total content of cadmium; Cr – total content of chromium; n.d. – not detected.

**Table S2**. The statistical significance of the differences between averages of tested parameters. The same letter means no significant differences between the values at the level of significance α = 0.05, one-way ANOVA variance analysis, Tukey's HSD test. Analysis performed separately for each variant of soil with digestate concentration as the differentiating factor.

|  | **P_c_**  **(%)** | **BD**  **(g·cm^-3^)** | **S**  **(m^2^·g^-1^)** | **SSA**  **(m^2^·g^-1^)** | **V_t_**  **(cm^3^·g^-1^)** | **D_av_**  **(nm)** | **σ_max_**  **(N)** | **YM**  **(MPa)** |
| --- | --- | --- | --- | --- | --- | --- | --- | --- |
| **Soil A** | 33.7^a^ | 1.68^f^ | 2.28^a^ | 1.55^c^ | 0.21^a^ | 2080.6^a^ | 26.7^b^ | 24.1^bc^ |
| **A+P 1%** | 33.6^a^ | 1.63^e^ | 2.39^ab^ | 1.56^c^ | 0.21^a^ | 2081.7^a^ | 31.7^bc^ | 31.9^d^ |
| **A+P 3%** | 35.7^ab^ | 1.57^d^ | 2.71^ab^ | 1.29^b^ | 0.23^ab^ | 2243.6^a^ | 51.5^d^ | 24.1^bc^ |
| **A+P 5%** | 37.1^bc^ | 1.50^c^ | 2.93^ab^ | 1.27^b^ | 0.25^b^ | 2575.7^a^ | 35.8^c^ | 25.2^cd^ |
| **A+P 10%** | 37.9^bc^ | 1.37^b^ | 3.29^ab^ | 1.00^a^ | 0.29^c^ | 3808.0^b^ | 28.3^bc^ | 17.0^ab^ |
| **A+P 15%** | 40.0^c^ | 1.26^a^ | 3.99^b^ | 1.24^b^ | 0.31^c^ | 3843.4^b^ | 14.6^a^ | 8.8^a^ |
| **Soil B** | 28.1^a^ | 1.78^e^ | 0.95^a^ | 0.47^a^ | 0.16^a^ | 8923.2^a^ | 6.4^a^ | 6.1^a^ |
| **B+P 1%** | 29.2^a^ | 1.74^de^ | 0.95^a^ | 0.44^a^ | 0.17^a^ | 8764.5^a^ | 7.6^a^ | 6.1^a^ |
| **B+P 3%** | 31.9^ab^ | 1.72^d^ | 1.43^a^ | 0.30^a^ | 0.19^a^ | 8509.1^a^ | 13.7^b^ | 8.0^ab^ |
| **B+P 5%** | 33.0^abc^ | 1.65^c^ | 1.49^a^ | 0.41^a^ | 0.20^ab^ | 8164.4^a^ | 15.1^b^ | 9.5^b^ |
| **B+P 10%** | 35.5^bc^ | 1.57^b^ | 3.01^b^ | 0.26^a^ | 0.24^bc^ | 9241.2^a^ | 25.9^c^ | 8.5^ab^ |
| **B+P 15%** | 37.5^c^ | 1.46^a^ | 3.32^b^ | 0.53^a^ | 0.26^c^ | 11523.8^b^ | 27.2^c^ | 13.2^c^ |
| **Soil C** | 31.7^a^ | 1.61^cd^ | 1.22^a^ | 0.27^ab^ | 0.19^a^ | 9130.4^d^ | 4.6^a^ | 6.5^a^ |
| **C+P 1%** | 32.1^a^ | 1.63^d^ | 1.66^a^ | 0.32^b^ | 0.20^a^ | 8704.3^c^ | 8.2^ab^ | 10.7^b^ |
| **C+P 3%** | 32.3^a^ | 1.60^cd^ | 1.80^a^ | 0.24^a^ | 0.20^a^ | 6940.9^a^ | 10.6^b^ | 4.0^a^ |
| **C+P 5%** | 32.5^a^ | 1.59^c^ | 2.35^a^ | 0.35^b^ | 0.20^a^ | 7052.5^a^ | 19.5^c^ | 5.2^a^ |
| **C+P 10%** | 35.8^b^ | 1.50^b^ | 2.16^a^ | 0.32^b^ | 0.25^b^ | 8109.0^b^ | 36.1^d^ | 19.5^c^ |
| **C+P 15%** | 39.4^c^ | 1.42^a^ | 2.89^a^ | 0.51^c^ | 0.28^c^ | 8305.1^b^ | 48.8^e^ | 13.2^b^ |
| **Soil D** | 35.1^a^ | 1.67^f^ | 5.23^a^ | 6.78^c^ | 0.22^a^ | 1321.7^a^ | 158.7^d^ | 86.1^b^ |
| **D+P 1%** | 36.5^a^ | 1.60e | 4.78^a^ | 4.69^b^ | 0.23^ab^ | 1403.0^a^ | 108.9^c^ | 76.4^b^ |
| **D+P 3%** | 37.3^a^ | 1.54^d^ | 4.31^a^ | 2.50^a^ | 0.25^bc^ | 1537.2^a^ | 93.7^c^ | 75.1^b^ |
| **D+P 5%** | 37.4^ab^ | 1.47^c^ | 4.93^a^ | 2.47^a^ | 0.26^c^ | 1748.4^ab^ | 85.1^bc^ | 40.2^a^ |
| **D+P 10%** | 38.8^ab^ | 1.39^b^ | 5.06^a^ | 2.19^a^ | 0.28^d^ | 2152.8^b^ | 58.4^ab^ | 36.5^a^ |
| **D+P 15%** | 41.4^b^ | 1.29^a^ | 5.54^a^ | 1.92^a^ | 0.34^e^ | 3209.8^c^ | 40.1^a^ | 23.3^a^ |
| **Soil E** | 24.0^a^ | 1.86^e^ | 3.76^a^ | 4.29^b^ | 0.13^a^ | 5784.5^a^ | 90.6^b^ | 69.9^b^ |
| **E+P 1%** | 26.9^b^ | 1.83^e^ | 3.74^a^ | 2.96^ab^ | 0.15^b^ | 5749.7^a^ | 76.0^ab^ | 39.8^a^ |
| **E+P 3%** | 27.9^b^ | 1.76^d^ | 4.33^a^ | 1.15^a^ | 0.16^b^ | 6128.8^a^ | 77.1^ab^ | 40.4^a^ |
| **E+P 5%** | 32.0^c^ | 1.68^c^ | 3.95^a^ | 1.13^a^ | 0.19^c^ | 7343.5^b^ | 69.2^a^ | 38.7^a^ |
| **E+P 10%** | 34.5^c^ | 1.61^b^ | 4.96^a^ | 0.99^a^ | 0.22^d^ | 7903.7^b^ | 67.7^a^ | 30.6^a^ |
| **E+P 15%** | 38.0^d^ | 1.50^a^ | 4.94^a^ | 1.08^a^ | 0.27^e^ | 9249.5^c^ | 68.5^a^ | 26.3^a^ |
| **Soil F** | 39.2^ab^ | 1.50^f^ | 3.68^a^ | 2.06^a^ | 0.27^a^ | 1732.7^a^ | 122.8^d^ | 77.9^e^ |
| **F+P 1%** | 39.6^ab^ | 1.46^e^ | 3.84^a^ | 1.88^a^ | 0.27^a^ | 2123.2^b^ | 83.1^c^ | 57.4^d^ |
| **F+P 3%** | 37.8^a^ | 1.40^d^ | 3.97^a^ | 1.65^a^ | 0.28^a^ | 2299.0^bc^ | 68.9^bc^ | 50.4^cd^ |
| **F+P 5%** | 37.8^a^ | 1.34^c^ | 4.48^a^ | 1.64^a^ | 0.29^ab^ | 2615.1^cd^ | 54.9^ab^ | 41.8^bc^ |
| **F+P 10%** | 38.7^ab^ | 1.24^b^ | 5.80^b^ | 1.52^a^ | 0.31^bc^ | 2633.3^cd^ | 48.1^a^ | 26.7^ab^ |
| **F+P 15%** | 40.9^b^ | 1.21^a^ | 6.92^b^ | 1.60^a^ | 0.33^c^ | 2892.5^d^ | 47.4^a^ | 17.7^a^ |

Abbreviation: P – digestate; A-F – types of soil; BD – bulk density; S – surface area of pores; SSA – specific surface area; D_av_ – average pore diameter; V_t_ – the total volume of pores, P_c_ – total porosity; σ_max_ – maximum strength of aggregate breakage; YM – Young’s modulus.
